# Metagenomics and metabolomics analysis to investigate the effect of Shugan decoction on intestinal microbiota in irritable bowel syndrome rats

**DOI:** 10.3389/fmicb.2022.1024822

**Published:** 2022-11-21

**Authors:** Lu Hang, Enkang Wang, Ya Feng, Yan Zhou, Yangyang Meng, Fengru Jiang, Jianye Yuan

**Affiliations:** Institute of Digestive Diseases, Longhua Hospital, Shanghai University of Traditional Chinese Medicine, Shanghai, China

**Keywords:** Shugan decoction, IBS-D, intestinal microbiota, metabonomics, metagenomics

## Abstract

**Background:**

The effect of Shugan Decoction (SGD) on intestinal motility and visceral hypersensitivity in Water avoid stress (WAS)-induced diarrhea predominant irritable bowel syndrome (IBS-D) model rats has been confirmed. However, the mechanisms of its action involved in the treatment of IBS-D need to be further studied. Intestinal microbiota plays an important role in maintaining intestinal homeostasis and normal physiological function. Changes in the intestinal microbiota and its metabolites are thought to participate in the pathophysiological process of IBS.

**Aim:**

This study aimed to analyze the influence of SGD on intestinal microbiota and fecal metabolites in IBS-D rats by multiple omics techniques, including metagenomic sequencing and metabolomics.

**Methods:**

We measured the intestinal motility and visceral sensitivity of three groups of rats by fecal pellets output and colorectal distension (CRD) experiment. In addition, metagenome sequencing analysis was performed to explore the changes in the number and types of intestinal microbiota in IBS-D model rats after SGD treatment. Finally, we also used untargeted metabolomic sequencing to screen the metabolites and metabolic pathways closely related to the therapeutic effect of SGD.

**Results:**

We found that compared with the rats in the control group, the fecal pellets output of the rats in the WAS group increased and the visceral sensitivity threshold was decreased (*P* < 0.05). Compared with the rats in the WAS group, the fecal pellets output of the SGD group was significantly decreased, and the visceral sensitivity threshold increased (*P* < 0.05). Besides, compared with the rats in the WAS group, the relative abundance of *Bacteroidetes* increased in SGD group, while that of *Firmicutes* decreased at the phylum level, and at the species level, the relative abundance of *Bacteroides sp. CAG:714*, *Lactobacillus reuteri* and *Bacteroides Barnesiae* in SGD group increased, but that of *bacterium D42-87* decreased. In addition, compared with the WAS group, several metabolic pathways were significantly changed in SGD group, including Taurine and hypotaurine metabolism, Purine metabolism, Sulfur metabolism, ABC transporters, Arginine and proline metabolism and Bile secretion.

**Conclusion:**

SGD can regulate specific intestinal microbiota and some metabolic pathways, which may explain its effect of alleviating visceral hypersensitivity and abnormal intestinal motility in WAS-induced IBS-D rats.

## Introduction

Irritable bowel syndrome (IBS) is a functional bowel disorder manifested in abdominal pain, abdominal distention, changed bowel habits and fecal appearance (Botschuijver et al., 2017), and its symptoms tend to be persist and recurrent (Shariati et al., 2019). According to the latest Roman IV criteria, the global prevalence of this disorder is about 4.1% (Sperber et al., 2021). At present, IBS can be roughly divided into four types: diarrhea predominant IBS (IBS-D), constipation predominant IBS (IBS-C), mixed IBS (IBS-M) and unsubtyped IBS (IBS-U) (Ford et al., 2017). Notably, IBS-D is the most common subtype (Oka et al., 2020). Although IBS is not a fatal disease, it seriously affects the life, study and work of patients, and also causes a serious economic burden to the society (Sebastián Domingo, 2022). Therefore, how to effectively prevent and treat IBS is an important problem to be solved urgently.

So far, the pathogenesis of IBS is unclear. The known pathophysiological mechanisms of IBS mainly include gastrointestinal motility abnormalities, visceral hypersensitivity, intestinal barrier dysfunction, intestinal microbiota disorders, *etc*. In recent years, more and more studies have confirmed that visceral hypersensitivity and intestinal dysmotility caused by enteric dysbacteriosis are the important pathological basis of IBS (Pimentel and Lembo, 2020). 5-hydroxytryptamine (5-HT) mediation is one of the pathways by which intestinal microbiota play its role in gastrointestinal function and the disturbances or disorders of 5-HT signal can induce IBS-like symptoms (Mishima and Ishihara, 2021; Murciano-Brea et al., 2021).

Treating IBS with traditional Chinese medicine can not only effectively improve its clinical symptoms, but also has the advantages of low cost and relatively small toxic and side effects (Teschke et al., 2015). Traditional Chinese medicine compound Shugan decoction (SGD) is an empirical prescription based on the traditional Chinese medicine syndrome differentiation of IBS-D patients in modern society. The formula consists of *white Atractylodes macrocephala*, *paeony root*, *tangerine peel*, *parsnip* and *Bupleurum*. Early studies confirmed that SGD can improve abdominal pain, diarrhea, defecation changes and other single symptoms in patients with IBS-D of liver spleen disharmony type, and the total effective rate can reach 86.67% (Xie et al., 2004). Further research found that SGD was more effective than Dicetel in relieving flatulence, and had better safety and tolerance (Pan and Xie, 2006). The latest randomized controlled clinical trial shows that SGD can not only effectively improve symptoms such as diarrhea, abdominal distension and bowel ringing, but also effectively relieve patients’ anxiety, depression and other mental states (Lu L. et al., 2020). Animal experiments showed that SGD could improve visceral hypersensitivity and gastrointestinal motility abnormalities in Water avoid stress (WAS)-induced IBS-D model rats by regulating 5-HT content and SERT expression in colon tissue (Shi et al., 2015; Lu et al., 2018). In recent years, it has been proved that SGD may regulate intestinal microbiota, thereby affecting intestinal 5-HT synthesis to improve symptoms such as abdominal pain and diarrhea (Shi et al., 2015).

In this study, metagenomic sequencing and untargeted metabolomics analysis were performed on rat feces to observe the effects of SGD treatment on intestinal microbiota and specific metabolic pathways in WAS-induced IBS model rats. On this basis, we aim to elucidate the role of SGD in IBS-D model rats on specific intestinal microbiota and the regulation of endogenous metabolites.

## Materials and methods

### Agents and materials

The components of SGD, i.e., *White attractylodes rhizome* (Baizhu) (Shang Hai De Hua GuoYao; Lot number: 2018061101), *white peony roo*t (Baishao) (Shang Hai Hua Pu Zhong Yao; Lot number: 2018042901), *dried old orange peel* (Chenpi) (Shang Hai Lei Yun Shang Zhong Yao; Lot number: 1805037), *ledebouriella root* (Fangfeng) (Shang Hai Yu Tian Cheng Zhong Yao; Lot number:2017022706), and *Radix bupleuri* (Chaihu) (Ma Chen Jiu Zhou; Lot number: E2018050101), were purchased as crude herbs from JinKe Pharmacy (Shanghai, China). Saikosaponin A (National Institute for Food and Drug Control; Lot number: 110777-201912), paeoniflorin (National Institute for Food and Drug Control; Lot number: 110736-201943), 5-O-Methylvisammioside (National Institute for Food and Drug Control; Lot number: 111523-201811), hesperidin (National Institute for Food and Drug Control; Lot number: 110721-201818), and cimicifugoside (National Institute for Food and Drug Control; Lot number: 111522-201913) were purchased from Shanghai Zhaorui Biological Technology Co. (Shanghai, China).

### Preparation of Shugan decoction extract

The quality ratios of White attractylodes rhizome (Baizhu), white peony root (Baishao), dried old orange peel (Chenpi), ledebouriella root (Fangfeng), and Radix bupleuri (Chaihu) are 6:4:3:4:6. SGD extract was prepared by decoction and water extraction in the Herbal Chemistry Lab in Shanghai University of TCM. The extraction process has been described previously (Wang Y. et al., 2020): herbal pieces were first soaked in distilled water for 30 min, then they were boiled in 6 times of water for 1 h. Next, the mixture was filtrated with 4 layers of gauze, and the filtrate was collected. The procedure was repeated twice, and the filtrate was freeze-dried to obtain the powder. The steps of freeze-drying are as follows: First, we freeze the drug into a solid state, and then sublimate and dry it to remove the ice crystals in the drug by sublimation. Next, we desorb and dry it to evaporate some of the water remaining in the product at a higher temperature, so that the residual water can meet the requirements. Finally, the dried products are sealed and packaged under vacuum or filled with inert gas for storage.

### Analysis and identification of Shugan decoction by high-performance liquid chromatography

According to the procedure described in our previous study (Wang Y. et al., 2020): Saikosaponin A, paeoniflorin, 5-*O*-Methylvisammioside, hesperidin, and cimicifugoside was dissolved in methanol and obtained 1 mg/mL standard solution separately. 500 mg SGD extract power was weighed and dissolved in distilled water. After ultrasonic shock for 40 min, the SGD solution was fixed at a constant volume of 10 mL. Then, 1 mL solution was injected into the activated C_18_ column, eluted with 10 mL water, and then eluted with 10 mL methanol. The methanol eluent was collected, concentrated to dry, dissolved with 1 mL methanol, and 50.89 mg/mL SGD sample solution was obtained through 0.45 μm microporous membrane. The standard solution and the SGD sample solution were analyzed using the Dionex UltiMate™ 3000 RSLC nano system (Thermo Scientific, MA, USA) equipped with a Corona® ultra™CAD detector, Luna^®^ C18 Column (Phenomenex, 250 mm × 4.6 mm, 5 mm), and a data station with analytical software (CHROMELEON^®^). Mobile phases consisted of A-Purified water and B-acetonitrile. Gradient was set as follows: 0 min, 5% B; 35 min 65.5% B; 35.001 min, 100% B; 40 min, 100% B. Column temperature was set at 25°C, DAD detection wavelength: 203, 254, 366 nm.

### Animals and treatments

Thirty male Sprague-Dawley (SD) rats, weighing 200g ± 20g, provided by Shanghai Bikai Experimental Animal Co., Ltd. [production license No.: SCXK (Shanghai) 2018-0006], raised in the Experimental Animal Center of Shanghai University of TCM under the standard temperature (21–24°C), humidity (50% ± 5%), light and dark cycle (12 h/12 h), and they had free access to standard rat chow and tap water. All the experiments in this study are in accordance with the regulations of the Animal Ethics Committee of Shanghai University of TCM (No. PZSHUTCM190906001). All the experiments were carried out between 8:00 and11:00 AM to minimize potential confounding effects of diurnal variations.

After a week of adaptive feeding, rats were randomly divided into 3 groups (*n* = 10 in each group). SGD group: 10 days WAS and gavage with SGD (1.28 g/kg body weight, lyophilized powder dosage, once per day) since the 4th day; WAS group: 10 days WAS and gavage with the same dose of saline; Control group: gavage with the same dose of saline.

### Water avoidance stress

Refer to the method pioneered by Bradesi et al. and used in our previous studies (Bradesi et al., 2005), rats were placed on the platform (10 cm long, 8 cm wide, 8 cm high) which was fixed in the center of a organic glass pool (45 cm long, 25 cm wide, 25 cm high) filled with water (25°C) to suffer from WAS for 1 h every day in 10 consecutive days.

### Fecal pellets counting

As described before (Bradesi et al., 2006), fecal pellets output in the one hour of WAS were counted to assess colonic motility every day for 10 consecutive days.

### Colorectal distension

On the 10th day after WAS, the pressure threshold to induce abdominal withdrawal reflex (AWR) in rats was measured by colorectal distension (CRD) test. The methods were as previously described (Spence and Moss-Morris, 2007): a balloon (5 mm diameter and 1 cm long) with catheter (2 mm diameter) was inserted into the colorectum 1 cm above the anus. The catheter was fixed to the root of the rat tail with adhesive tape. Then the balloon was inflated gradually by one experimenter; the abdominal wall reactions of the rats were observed by another experimenter and a voice command was issued by him when the first AWR appeared; then the pressure value at this moment was recorded. Every two measurements were done with an interval of 3 min, and the average value was calculated after 3 times of measurement.

### Fecal sample collection

After SGD treatment and WAS, feces from the Control, WAS, and SGD groups were obtained under sterile conditions and stored at −80°C. The fecal samples were divided into two parts, one part was used to perform Metagenomics analysis, and another part was used for untargeted metabolomics analysis.

### Hematoxylin eosin staining

The colon tissues were fixed in 4% paraformaldehyde for 48 h after the luminal content was washed off with ice normal saline. Then the paraffin sections were made by dehydration, transparency, wax soaking, embedding and sectioning. Hematoxylin eosin (H&E) solution staining, neutral gum sealing, and observation under ordinary optical microscope (Nikon Corporation, Japan) were done in sequence.

### Metagenomics analysis

DNA was extracted from rat fecal samples, then microbial DNA was fragmented, metagenomic sequencing was performed based on Illumina NovaSeq high-throughput sequencing platform, Whole Genome Shotgun (WGS) strategy was adopted. The extracted metagenomic total DNA was randomly interrupted into short fragments and inserted fragment libraries of appropriate length were constructed. These libraries were paired with PE sequencing. FastQC was used to test data quality. MEGAHIT was used for metagenomic sequence splicing. Meta GeneMark^1^ was used for gene prediction and the identification of Open Reading Frame (ORF), the corresponding gene prediction files and protein sequences were obtained. The non-redundant protein sequence set was compared with the common protein database to annotate and analyze the gene function in each sample. QIIME (Quantitative Insights Into Microbial Ecology) software was used to obtain the relative abundance distribution table of each sample corresponding to each functional level of each database. By using the software MEGAN,^2^ each sample and taxonomy of species abundance information of data can map to NCBI Taxonomy provided by the microbial classification tree,^3^ which can be in a standard classification system, uniformly present the specific composition of all samples at each classification level. Next, with the help of the “random forest” toolkit of R software, the random forest algorithm is used to select the functions/species with significant differences in abundance distribution among different groups. Specifically, in order to compare the diversity of different samples, the abundance spectrum of underlying functional groups or the composition spectrum of species level annotated in each functional database of all samples were firstly randomly resampled according to the lowest sequencing depth (i.e., “sequence volume leveling”), so as to correct the diversity differences caused by sequencing depth. Subsequently, QIIME software was used to calculate four diversity indices including Shannon index for each sample. On the basis of the above analysis, we conducted Beta diversity analysis on the abundance spectrum of functional annotation and the composition spectrum of species annotation respectively, so as to investigate the differences between samples at the two levels of bacterial flora function and species composition. Mainly through three methods: Principal Component analysis (PCA), Multidimensional Scaling analysis (MDS) and Clustering analysis, the metagenomic multi-dimensional data structure was decomposed naturally and the samples were ordinated to observe the differences between samples. R software and QIIME software were used to perform PCA analysis on the abundance spectrum or species level composition spectrum of the underlying functional groups annotated in each functional database of metagenomic samples, and 2D and 3D images were used to describe the natural distribution characteristics among samples. QIIME software was used to map the first two- or three-dimensional data obtained from PCoA analysis, so as to know the spatial distribution characteristics of community samples based on metagenomic functional abundance spectrum or species composition spectrum, and quantify the size of differences between samples (groups). R software was used to perform NMDS analysis on the Bray-Curtis distance matrix obtained, and the structure distribution of community samples was described by two-dimensional ranking map. Using QIIME software, the Bray-Curtis distance matrix obtained was analyzed by UPGMA clustering and visualized by R software. According to the abundance spectrum or species-level composition spectrum of the underlying functional groups annotated in the functional database of each sample (group), R software was used to calculate the number of common taxa of each sample (group), and the number of common and unique functions/species of each sample (group) was visually presented by Venn diagram.

### Untargeted metabolomics analysis

The metabolites in feces were extracted and analyzed by UHPLC (Ultra high-performance liquid chromatography) platform of Shanghai Paiseno Technology Co., LTD. The specific steps of untargeted metabolomics mainly include: sample preparation, QC preparation, sample LC-MS/MS mass spectrometry, data analysis and experimental report, etc. In order to control the quality of this experiment, the researchers prepared QC samples at the same time, and QC samples were samples mixed with equal amounts of all samples. QC samples were used in the balanced chromatography-mass spectrometry system and the state of the instrument, and were used to evaluate the stability of the system throughout the experiment. After the liquid nitrogen was ground, 400 μl of pre-cooled methanol/acetonitrile/aqueous solution (4:4:2, V/V) was added to the sample, mixed by vortexing, stood at −20°C for 60 min, centrifuged at 14,000 *g* at 4°C for 20 min, and the supernatant was dried under vacuum. For mass spectrometry analysis, 100 μL acetonitrile aqueous solution (acetonitrile: Water = 1:1, v/v) were redissolved, vortexed, centrifuged at 14,000 *g* for 15 min at 4°C, and 2 μL of the supernatant was taken for sample analysis. The samples were separated on Agilent 1290 Infinity LC ultra-high performance liquid chromatography (UHPLC) HILIC column. The column temperature is 25°C; Flow rate 0.3 mL/min; Injection volume 2 μL; Mobile phase composition: A: water + 25 mM ammonium acetate + 25 mM ammonia, B: acetonitrile; The gradient elution procedure was as follows: 0–1 min, 95%b; 1–14 min, B changed linearly from 95 to 65%; 14–16 min, B changed linearly from 65 to 40%; 16–18 min, B maintained at 40%; 18–18.1 min, B changed linearly from 40 to 95%; 18.1– 23 min, B maintained at 95%; The samples were placed in an autosampler at 4°C during the whole analysis. Electrospray ionization (ESI) positive ion mode and negative ion mode were used for detection. The samples were separated by UHPLC and analyzed by mass spectrometry using a Triple TOF 6600 mass spectrometer (AB SCIEX). The ESI Source conditions after HILIC chromatographic separation were as follows: Ion Source Gas1 (Gas1): 60, Ion Source Gas2 (Gas2): 60, Curtain Gas (CUR): 30, Source temperature: 600°C, IonSapary Voltage Floating (ISVF) ± 5,500 V (both positive and negative modes); TOF MS Scan M/Z Range: 60–1,000 Da, Product ION Scan M/Z Range: 25–1,000 Da, TOF MS scan accumulation time is 0.20 s/spectra, Product ion scan accumulation time is 0.05 s/spectra; The secondary mass spectra were obtained using Information dependent acquisition (IDA) and their high sensitivity mode, Declustering potential (DP): *In situ*: ± 60 V (positive and negative modes), Collision Energy: 35 ± 15 eV, IDA non-frontiers within 4 Da, Candidate ions to monitor per cycle: 6. The final Data set was imported into SIMCA 16.0.2 software using an internal standard normalization method (Sartorius Stedim Data Analytics AB, Umea, Sweden; RRID:SCR_014688) was applied to principal component analysis (PCA) and orthogonal partial least squares discriminant analysis (OPLS-DA). One-dimensional statistical analysis including Student’s *t*-test and multiple variation analysis, R software was used to draw the volcano map. VIP > 1 and *P* value < 0.05 in OPLS-DA model were used as screening criteria, and then cluster analysis and KEGG metabolic pathway analysis were performed on the differentially expressed metabolites.

### Statistical analysis

SPSS version 25.0 (SPSS, Chicago, IL, USA) and GraphPad Prism 9.0 (La Jolla, CA, USA) were used for data analysis. Each value was expressed as mean ± SE. If data were subject to normality and homogeneity of variance, one-way analysis of variance (One-way ANOVA) and followed LSD-t test was use for analyzing the differences among the groups. If disobedient, the rank-sum test was used. *P* < 0.05 was considered statistically significant.

## Results

### Effects of Shugan decoction on irritable bowel syndrome model rats

**FIGURE 1:**
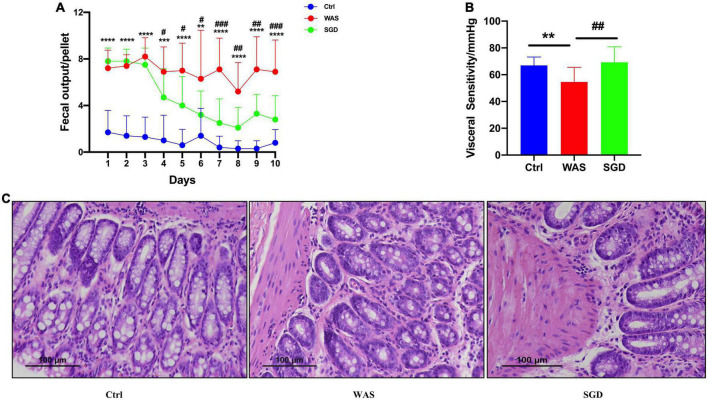
Effects of SGD on daily fecal pellets output, visceral sensitivity and colon histology in WAS-induced IBS-D model rats. **(A)** Daily fecal pellets output of rats. The daily fecal pellets output in the WAS group was higher than that in the control group, and SGD reduced daily fecal pellets output in the WAS group. **(B)** Visceral sensitivity in rats. The visceral sensitivity threshold of the WAS group was lower than that of the control group, and SGD improved the visceral sensitivity threshold of the rats in the WAS group. **(C)** Histology of the colon of the rats. H&E staining showed that there were no pathological changes in the colon tissues of rats in each group. Control group; WAS group; SGD + WAS group (*n* = 10 per group). Data are presented as mean ± standard deviation (*P < 0.05, **P < 0.01, ***P < 0.001, ****P < 0.0001; ^#^*P* < 0.05, ^##^*P* < 0.01, ^###^*P* < 0.001).

It was found that compared with control group, the amount of fecal pellets output of rats in WAS group was significantly increased. Compared with WAS group, the SGD group had reduced amount of fecal pellets output (*P* < 0.05, Figure 1A). In addition, we found that compared with control group, visceral sensitivity threshold of rats in WAS group was decreased. Compared with WAS group, visceral sensitivity threshold of rats increased in SGD group (*P* < 0.001, Figure 1B). No significant pathological changes were found in colonic mucosa in WAS and SGD groups, compared with control group (Figure 1C).

### Effects of Shugan decoction on intestinal microbiota of irritable bowel syndrome model rats

**FIGURE 2:**
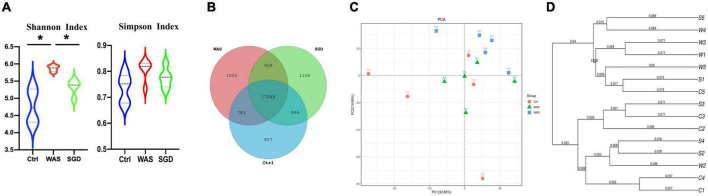
Effects of SGD on the intestinal microbiota of IBS-D model rats. **(A)** Shannon index and Simpson index were calculated after refining to an equal number of sequence reads for all samples. The Shannon index of the WAS group was higher than that of the control and SGD groups, while Simpson index has no significant difference among the three groups. **(B)** Venn diagram represented OTUs in each group. **(C)** PCA scores based on the weighted Unifrac index were different among groups. **(D)** The weighted Unifrac index based PCA score phylogenetic tree of gut microbiota. Control group; WAS group; SGD group (*n* = 5 per group) (*P < 0.05).

We used a sequencing platform to conduct metagenomic sequencing of rat fecal bacteria DNA, aiming to study the changes of intestinal microbiota species in IBS-D model rats before and after SGD treatment. The results showed that shannon index in WAS group was significantly higher than that in control and SGD group, while there is no significant difference among the three groups in Simpson index (Figure 2A). In addition, Venn diagram analysis indicated that the three groups shared 19,748 OTUs, with 857 OTUs peculiar to control group, 1,050 OTUs peculiar to WAS group and 1,166 OTUs peculiar to SGD group (Figure 2B). The results of principal coordinate analysis (PCA) and systematic clustering tree both manifested that the intestinal microbiota of the three groups were significantly different (Figures 2C,D).

**FIGURE 3:**
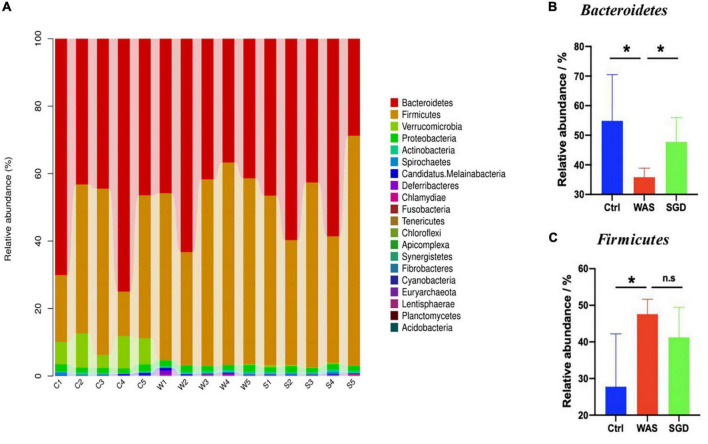
Effects of SGD on the intestinal microbiota of IBS-D model rats at the phylum level. **(A)** SGD treatment changed the intestinal microbiota at the phylum level. **(B)** The relative abundance of *Bacteroidetes* was decreased in the WAS group compared with the control group; compared with the WAS group, the relative abundance of *Bacteroidetes* increased in the SGD group. **(C)** Compared with the control group, the relative abundance of *Firmicutes* in the WAS group increased; compared with the WAS group, the relative abundance of *Firmicutes* in the SGD group decreased (*P < 0.05).

**FIGURE 4:**
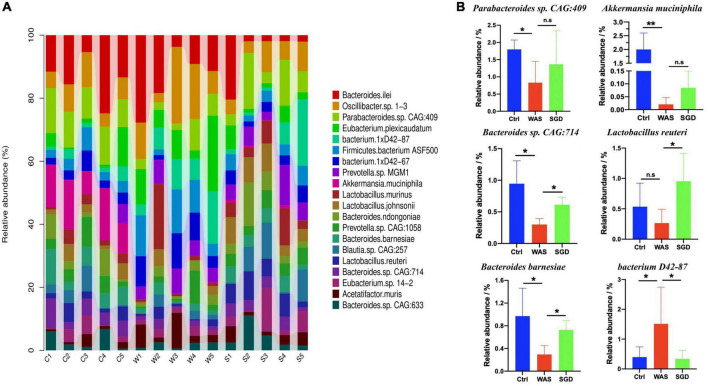
Effects of SGD on the intestinal microbiota of IBS-D model rats at the species level. **(A)** SGD treatment changed the intestinal microbiota at the species level. **(B)** Compared with the control group, the relative abundance of *Parabacteroides sp. CAG: 409*, *Akkermansia muciniphila, Bacteroides sp. CAG*: 714 and *Bacteroides barnesiae* decreased, and the relative abundance of *bacterium 1xD42-87* increased in the WAS group; compared with the WAS group, the relative abundance of *Bacteroides sp. CAG: 714*, *Lactobacillus reuteri*, and *Bacteroides barnesiae* increased and the relative abundance of *bacterium 1xD42-87* decreased in the SGD group. Control group; WAS group; SGD group (*n* = 5 per group) (*P < 0.05, **P < 0.01).

**FIGURE 5:**
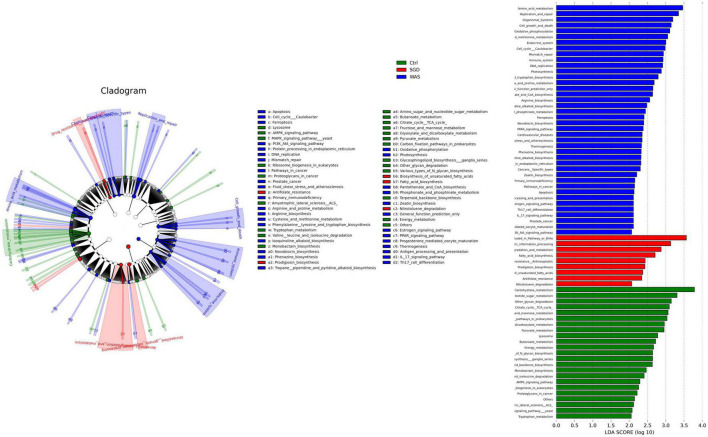
Effect of SGD on microbial function. LEfSe analysis shows significantly different function between the three groups **(left panel)** and function with significantly different abundances (LDA scores >2) **(right panel)**. LDA, linear discriminant analysis; LEfSe, LDA effect size.

Next, we studied the changes of intestinal microbiota and its abundance at phylum and species level in each group. Firstly, at the phylum level, 20 phyla could be found in each group (Figure 3A) and the most abundant phyla in each group were *Bacteroidetes* and *Firmicutes.* Compared with control group, the relative abundance of *Bacteroidetes* in WAS group decreased (*P* < 0.05, Figure 3B) and *Firmicutes* increased (*P* < 0.05, Figure 3C). Compared with WAS group, the relative abundance of *Bacteroidetes* in SGD group increased (*P* < 0.05, Figure 3B). Secondly, at the species level, we found that, compared to control group, the relative abundance of *Parabacteroides sp. CAG:409*, *Akkermansia muciniphila*, *Bacteroides sp. CAG:714* and *Bacteroides Barnesiae* decreased, and the relative abundance of *Bacterium D42-87* increased in WAS group. Compared with WAS group, the relative abundance of *Bacteroides sp. CAG:714*, *Lactobacillus reuteri* and *Bacteroides Barnesiae* increased, and the relative abundance of *Bacterium D42-87* decreased in SGD group (*P* < 0.05 and *P* < 0.01, Figures 4A,B). Besides, the possible function related to the differential intestinal microbiota has been analyzed. The results are shown in Figure 5.

**FIGURE 6:**
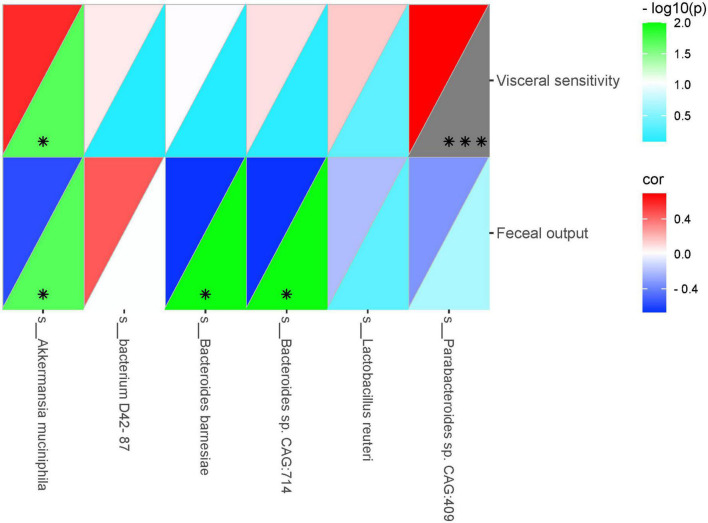
Heatmap showing correlations between significantly altered intestinal microbiota and rat phenotype parameters. Color intensity indicates the degree of correlation. The top left represents the correlation, and the bottom right represents –log10(*P*). Red, positive correlation; blue, negative correlation (*n* = 5 rats per group) (*P < 0.05, ^***^P < 0.001).

To further investigate whether the improvement of VH and intestinal motility by SGD is related to the effect of significantly altered intestinal microbiota, we conducted correlation analysis between these significantly changed intestinal strains and visceral sensitivity threshold and amount of fecal pellets output in rats, respectively. On this basis, the heatmap is used to further analyze the correlation between significantly altered intestinal microbiota and rat phenotype parameters. As illustrated in the correlation heatmap, the abundances of *Parabacteroides sp. CAG:409*, *Akkermansia muciniphila*, *Bacteroides sp. CAG:714*, *Bacterium D42-87, Lactobacillus reuteri* and *Bacteroides Barnesiae* were positively correlated with visceral sensitivity threshold. Moreover, except for *Bacterium D42-87*, the abundances of *Parabacteroides sp. CAG:409*, *Akkermansia muciniphila*, *Bacteroides sp. CAG:714*, *Lactobacillus reuteri* and *Bacteroides Barnesiae* were all negatively associated with the amount of fecal pellets output (Figure 6).

### Effects of Shugan decoction on the metabolites of intestinal microbiota in fecal of irritable bowel syndrome model rats

**FIGURE 7:**
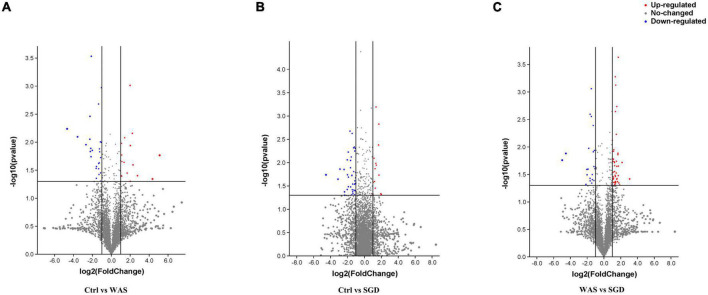
Metabolic profiling: volcano plot. **(A)** 26 metabolites (red dots) were significantly up-regulated in the feces of the WAS group compared with the control group; **(B)** 14 metabolites (red dots) were significantly up-regulated in the feces of the SGD group compared with the control group; **(C)** 44 metabolites (red dots) were significantly up-regulated in the feces of the SGD group compared with the WAS group. The red dots in the figure indicate FC > 2.0 and *P* < 0.05. The differential metabolites were screened by univariate statistical analysis.

Untargeted metabolomics analysis was performed by ultra-high performance liquid chromatography-Q-TOF MS. Volcanic map showed that there were 26 significantly upregulated metabolites in WAS group (The red dots in the figure are metabolites with FC > 2.0 and *P* value <0.05, that is, the difference metabolites screened by univariate statistical analysis) compared with control group (Figure 7A), 14 metabolites were significantly up-regulated in the feces of the SGD group compared with the control group (Figure 7B), and 44 significantly upregulated metabolites in SGD compared with the WAS group (Figure 7C).

**FIGURE 8:**
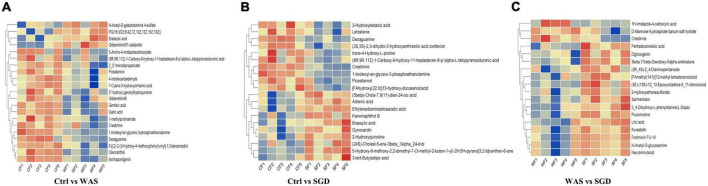
Metabolic profiling: hierarchical clustering results. **(A)** Significantly different metabolites between control group and WAS group. **(B)** Significantly different metabolites between control group and SGD group. **(C)** Significantly different metabolites between WAS group and SGD group. The abscissa represents different samples, and the ordinate represents significantly different metabolites.

In addition, we performed cluster analysis on all metabolites detected and constructed a heat map (Figure 8). The results showed that, compared with control group, *N*-acetyl-D-Galactosamine 4-sulfate, PG (18:0/22:6 (4Z,7Z,10Z,13Z,16Z,19Z), Brassylic acid, gibberellinA51-catabolite were significantly up-regulated in WAS group, while 5-amino-4-imidazolecarboxylate, (8R,9R,11Z)-1-carboxy-9-hydroxy-11-heptadecen-8-yl alpha- L-talopyranosiduronic acid, 2_2-iminodipropanoate, Piceatan-nol, 4-indolecarbaldehyde, ?-Cyano-3-hydroxycinnamic acid, 3″-hydroxy-geranylhydroquinone, GibberellinA8, Gentisic acid, Gallic acid, 1-methylProlinamide, Creatinine, 1-Linoleoyl-sn-glycero-3-phosphoethanolamine, Dezaguanine, 5-[(Z)-2-(3-hydroxy-4-methoxyphenylvinyl-1,3-benzenediol, Oleocanthal, Isorhapontigenin decreased significantly (Figure 8A). In addition, compared with control group, the 3-Hydroxysebacic acid, Letosteine, Dezaguanine, (2S,3S)-2,3-dihydro-3-hydroxyanthranilic acid zwitterion, trans-4-Hydroxy-L-proline, (8R,9R,11Z)-1-Carboxy-9-hydroxy-11-heptadecen-8-yl alpha-L-talopy- ranosiduronic acid, Creatinine, 1-linoleoyl-sn-glycero-3-phosphoethanolamine, Piceatannol, [FAhydroxy(22:0)]13-hydroxy-docosanoicacid in SGD group decreased significantly, while (5beta)-Chola-7,9(11)-dien-24-oic acid, Adrenic acid, Ethylenediaminetetraacetic acid, Karwinaphthol B, Brassylic acid, Gynocardin, 2-Hydroxyquinoline, (4S)-Cholest-5-ene-3beta_7alpha_24-triol, 5-Hydroxy-8-methoxy-2,2-dimethyl-7-(3-methyl-2-buten-1-yl)-2H,6H-pyrano[3,2-blxanthen-6-one, 3-tert-Butyladipic acid were significantly up-regulated (Figure 8B). Besides, compared with WAS group, the levels of 1H-lMidazole-4-carboxylic acid, D-Mannose 6-phosphate barium salt hydrate and Creatinine were significantly decreased, while Pentadecanedioic acid, Digitoxigenin, 3beta, 17beta-diacetoxy-5alpha-androstane, 2R_4S-2_4-Diaminopentanoate, [FAmethyl (14:O)]12-methyl-tetradecanoicacid, (9Z)-(13S)-12_13-Epoxyoctadeca-9_11-dienoicacid, 2-Hydroxyethanesulfonate, Sarmentosin, 3_4-Dihydroxy-L-phenylalanine (L-DOPA), Fluocinolone, Uric acid, Fluvastatin, Fortimicin FU-10, *N*-Acetyl-D-glucosamine and Neuraminicacid increased significantly in SGD group (Figure 8C).

**FIGURE 9:**
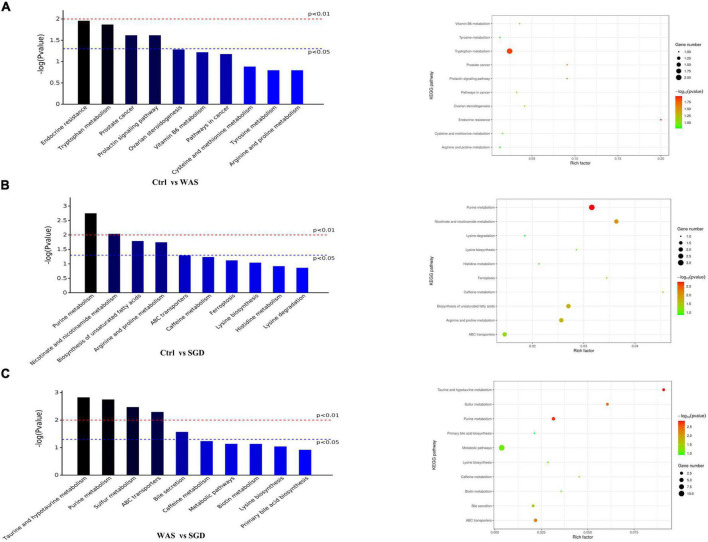
Metabolic profiling: KEGG pathway. **(A)** KEGG pathways with significant differences between control and WAS groups. **(B)** KEGG pathways with significant differences between control and SGD groups. **(C)** KEGG pathways with significant differences between WAS and SGD groups. (Left) Histogram of enriched KEGG pathways statistics. The *x*-axis indicates KEGG metabolic pathways with significant differences and the *y*-axis indicates the *P* value of each KEGG pathway. (Right) Bubble chart of enriched KEGG pathways statistics. Rich factor is the ratio of the differentially expressed gene number to the total gene number in a certain pathway. The color and size of the dots represent the range of the –log(*P*-value) and the number of genes mapped to the indicated pathways, respectively. pathways that *P* < 0.05 are shown in the figure.

Finally, Bioinformatics analysis showed that compared to control group, the metabolic pathways with significant differences in WAS group were as follows: endocrine resistance, tryptophan metabolism, prostate cancer and prolactin signaling pathway (Figure 9A and Table 1). In addition, compared to control group, the metabolic pathways that were significantly different in SGD group were as follows: purine metabolism, nicotinate and nicotinamide metabolism, biosynthesis of unsaturated fatty acids, and arginine and proline metabolism (Figure 9B and Table 1). What’s more, compared to WAS group, the metabolic pathways with significant differences in SGD group were as follows: taurine and hypotaurine metabolism, purine metabolism, sulfur metabolism, ABC transporters and bile secretion (Figure 9C and Table 1).

**TABLE 1:** KEGG pathway with significant differences between different groups are as follows (*P* < 0.05).

Groups (A vs B)	KEGG metabolic pathways’ name	*P* value
Ctrl vs WAS	Endocrine resistance	0.011
	Tryptophan metabolism	0.0135
	Prostate cancer	0.0241
	Prolactin signaling pathway	0.0241
Ctrl vs SGD	Purine metabolism	0.00178
	Nicotinate and nicotinamide metabolism	0.00914
	Biosynthesis of unsaturated fatty acids	0.0162
	Arginine and proline metabolism	0.0179
WAS vs SGD	Taurine and hypotaurine metabolism	0.00149
	Purine metabolism	0.00178
	Sulfur metabolism	0.00336
	ABC transporters	0.00506
	Bile secretion	0.0269

## Discussion

In this study, the results showed that compared with control group, the amount of fecal pellets output in WAS group was significantly increased, accompanied by an increase in visceral sensitivity, and there were no pathological changes in colonic epithelial tissues, indicating that the model was successfully established. Consistent with previous study (Shang et al., 2013; Wang Y. et al., 2020), SGD reduced the amount of fecal pellets output of WAS rats and restored their visceral sensitivity.

Next, we conducted metagenomic sequencing of fecal bacteria DNA in each group to study changes in microbial composition. The results showed that compared with the control group, the α-diversity of the intestinal microbiota of the rats in the WAS group was increased, mainly manifested as a significant increase in the shannon index. In addition, both PCA analysis and phylogenetic clustering tree showed significant differences among the groups, indicating that the β-diversity of the intestinal microbiota was significantly different among the three groups. On this basis, when we studied the changes of intestinal microbiota at the phylum level, we found that compared with the control group, the relative abundance of *Bacteroidetes* in the WAS group was significantly lower, which was consistent with previous research results (Jacobs et al., 2021). *Bacteroides* is a kind of beneficial bacteria and is considered to be the main synthesizer of vitamin K. what’s more, it can maintain host intestinal homeostasis by regulating the level of short-chain fatty acids (SCFAs) (Nagpal et al., 2018), and it may also reduce the production of lipopolysaccharide in intestinal microorganisms, inhibiting inflammatory response (Yoshida et al., 2018). At last, it can not only down-regulate the level of interleukin-6 (IL-6), but also up-regulate the expression of occludin, playing an important role in the treatment of antibiotic-associated diarrhea (Guo et al., 2021). Existing studies have pointed out that *Bacteroides* in the rectal mucosa can be used as a microbial marker to distinguish patients with IBS-D from normal people (Zhu et al., 2021). Interestingly, we observed increased abundance of *Bacteroidetes* after administration of SGD. This suggests that SGD may improve the diarrhea symptoms of IBS patients by increasing the abundance of *Bacteroidetes*, but the specific mechanisms need to be studied further. As sequencing technology continues to improve, we have been able to observe changes in intestinal microbiota at the species level. As mentioned above, several strains in WAS group changed significantly compared with control group. Regarding them, it has been found that *Parabacteroides* can produce acetate to reduce the infiltration of neutrophils, which plays a role in acute pancreatitis (Lei et al., 2021). *Akkermansia Muciniphila* can directly regulate the integrity of host intestinal epithelial cells and the thickness of mucus layer to promote intestinal health. In addition, its metabolite propionic acid can bind with G protein-coupled receptor (GPR) 43 to mediate changes in downstream pathways, thus playing a key role in immunomodulatory, and is closely related to metabolic diseases such as metabolic syndrome (Zhang et al., 2019). What’s more, *Akkermansia muciniphila* was also negatively correlated with pain (Cruz-Aguliar et al., 2019). Interestingly, *Lactobaccillus Reuteri* is mainly used for the treatment of IBS-C, functional abdominal pain or constipation related diseases (Pärtty et al., 2018; Hojsak, 2019). As a kind of probiotics, it can not only regulate intestinal microbiota to relieve the symptoms of gastroenteritis patients, but also promote intestinal movement to relieve chronic constipation (Saviano et al., 2021). *Bacteroides Barnesiae* is mainly related to immune function (Su et al., 2021). Notably, the abundance of these significantly reduced intestinal microbiota recovered after the administration of SGD. Therefore, we speculated that increasing in the abundance of these strains was closely related to the relief of abdominal pain and diarrhea symptoms after SGD administration. In the future, we should conduct further studies on these strains to develop new biomarkers and/or probiotics for the diagnosis and treatment on IBS.

On this basis, we conducted a metabolomics study on rat fecal samples to screen out metabolites with statistical and biological significance, clarify the mechanism of metabolic process and expression changes in IBS model rats, and further explore the correlation between them. For example, whether they are in the same metabolic pathway, or whether they are upstream and downstream metabolites. When analyzing the differential metabolites between two groups of samples, Volcano Plot, as a univariate analysis method, can intuitively show the significance of metabolite changes. It helped us to screen metabolites as potential markers. We found that 26 metabolites were significantly up-regulated in the WAS group compared with the control group, and 44 metabolites were significantly up-regulated in the SGD group compared with the WAS group. After observing this phenomenon, we conducted Hierarchical Clustering for each group of samples, so as to accurately screen out marker metabolites and study the changes of related metabolic processes. The results revealed that most of the significantly changed metabolites in the WAS group showed a downward trend compared with the control group. Among them, Oleocanthal can act as an anti-inflammatory agent, a heat shock protein (HSP) 90 inhibitor, a cyclooxygenase (COX)1 and 2 inhibitor and an antioxidant. Dezaguanine is a purine nucleoside analog with antitumor and viral activity. While Gallic acid is mainly used in veterinary medicine as a bowel astringent and antidiarrheal. In addition, Gentisic acid is a metabolite of human salicylic acid, which is associated with the occurrence and development of colorectal cancer (Brown et al., 2016). Notably, most of the significantly changed metabolites in the SGD group showed an upward trend compared with the WAS group. N-Acetyl-D-glucosamine is related to amino acid metabolism pathway and is involved in the occurrence and development of colon cancer (Brown et al., 2016; Sinha et al., 2016) and diverticulum-related diseases (Tursi et al., 2016). Fluvastatin is a commonly used cholesterol-lowering agent, which can act by inhibiting 3-hydroxy-3-methyl glutaryl coenzyme A reductase (HMGR), and associated with abdominal pain, anorexia (Li et al., 2016), indigestion (Greten et al., 1994) and other digestive system diseases. Besides, uric acid (UA) is the main antioxidant in human plasma, which can inhibit or delay the oxidation reaction, and is related to diseases such as acute kidney injury and colorectal cancer (Wang et al., 2017). Fluocinolone is an anti-inflammatory glucocorticoid. Levodopa, an amino acid precursor of dopamine, is associated with aromatic L-amino acid decarboxylase deficiency (Abdenur et al., 2006). It can cross the blood-brain barrier through various pathways and decarboxylate to form dopamine. Pentadecanedioic acid, as a long-chain fatty acid, is the basic component of phospholipids, triglycerides and cholesterol, as well as the main substrate in energy metabolic reactions, and is closely related to metabolic syndrome such as obesity, hypertension and dyslipidemia (Wang L. et al., 2020). By analyzing these metabolites, we can see that WAS can cause a decrease in the expression of metabolites related to anti-inflammatory, antioxidant, antidiarrheal, and anti-tumor, and SGD administration can increase the expression of some specific metabolites to treat abdominal pain, indigestion, colon cancer and other digestive system diseases.

There is a certain relationship between the significantly changed intestinal microbiota and the differentially expressed metabolites. Existing studies have found that N-Acetyl-D-glucosamine is indispensable for the growth of Akkermansia muciniphila (Ouwerkerk et al., 2016; Ropot et al., 2020). In addition, UA is the end-product of purine metabolism in the liver, and when purine metabolism is impaired, serum UA levels will increase, further forming hyperuricemia, which will eventually lead to gout. However, Lactobacillus reuteri can stabilize serum uric acid level and prevent hyperuricemia (Kuo et al., 2021). Other studies have pointed out that the abundance of Akkermansia mucinphila in the intestine is related to the levels of uric acid and xanthine, and plays an important role in fatty acid synthesis and energy metabolism (Lu C. et al., 2020; Han et al., 2021; Liao et al., 2022).

**FIGURE 10:**
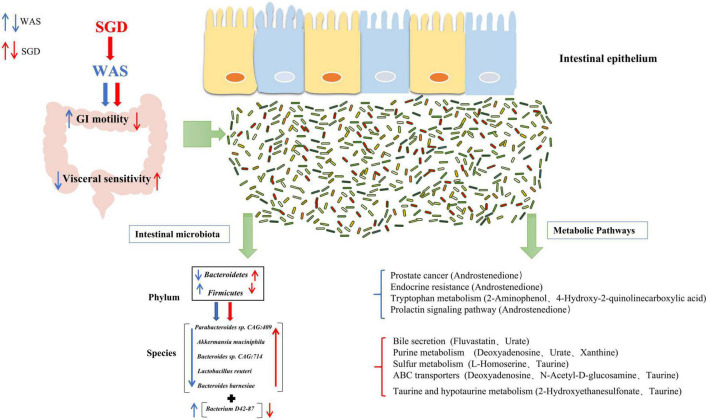
Effect of SGD on intestinal microbiota in IBS-D rats.

After screening out these obviously different metabolites, cluster analysis and KEGG metabolic pathway analysis on them were performed and we obtained the metabolic pathways with significant differences in each group of rats (important metabolites in this pathway are in brackets). Compared with control group, there were remarkable differences in endocrine resistance (Androstenedione), tryptophan metabolism pathways (2-Aminophenol, 4-Hydroxy-2-quinolinecarboxylic acid), prostate cancer (Androstenedione) and Prolactin signaling pathway (Androstenedione) in WAS rats. To begin with, endocrine resistance is mainly related to colon function. Some studies have found that IL-6, a pro-inflammatory cytokine, is significantly elevated in patients with IBS, which can regulate intestinal secretion and participate in the development of IBS (O’Brien et al., 2021). Regarding the Tryptophan metabolism pathway, studies have found that the decreased serum tryptophan concentration exhibited by SERT ^–/–^ rats is associated with visceral hypersensitivity and abnormal gastrointestinal motility (Bi et al., 2021). In addition, the tryptophan metabolic pathway is considered to be one of the main metabolic pathways in the WAS-induced IBS model rats, and the changes in the intestinal microbiota of the model rats are closely related to the changes in tryptophan metabolism (Mishima and Ishihara, 2021). Recent studies indicate that dysregulation of tryptophan/serotonin metabolism in feces and serum is closely related to the severity of IBS. The kynurenine pathway is thought to be the main pathway for the metabolism of L-tryptophan (Fila et al., 2021; Han et al., 2022). 2-Aminophenol (2AP) is the structural precursor of L-3-hydroxy kanurine (L-3HOK) and 3-hydroxy anthranilic acid (3HAA), whose oxidative autodimerization can induce neurotoxicity of Kynurenines, the oxidative degradation products of tryptophan (Zhuravlev et al., 2018). We can see that Androstenedione is the major metabolite of both Prostate cancer and Prolactin signaling pathway. Existing studies suggest that they are related to prostate cancer (Hettel et al., 2018) and adrenal cortical function (Kaminska et al., 2002), respectively. Compared with WAS group, SGD group showed significant differences in metabolic pathways such as taurine and hypotaurine metabolism (2-Hydroxyethanesulfonate, Taurine), purine metabolism (Deoxyadenosine, Urate, Xanthine), sulfur metabolism (L-Homoserine, Taurine), ABC transporters (Deoxyadenosine, *N*-Acetyl-D-glucosamine, Taurine) and bile secretion (Fluvastatin, Urate). Among them, taurine and hypotaurine metabolism can play an important role in bile acid metabolism pathway, leading to intestinal microbiota disturbance by inhibiting the growth of beneficial bacteria and causing intestinal inflammation (Schliess et al., 1997; Cerdó et al., 2018). Studies have pointed out that its important metabolite Taurine is involved in the occurrence and development of chronic transport constipation, UC and colorectal cancer (Liu et al., 2019; Zhou et al., 2019; Zhu et al., 2022). Purine metabolism is linked to the occurrence and development of colitis (Wu et al., 2020) and type 2 diabetes (Zhao et al., 2020). Interestingly, rhein, as a major metabolite in Purine metabolism, can reduce uric acid levels and thus alleviate Dextran sulfate sodium salt (DSS)-induced chronic colitis (Wu et al., 2020). As for sulfur metabolism, studies have confirmed that the energy and lipid metabolism capacity of the gut microbes in WAS group decreased, while the fatty acid and sulfur metabolism capacity increased (Fourie et al., 2017). The marked increase in the content of exogenous hydrogen sulfide, a major component involved in sulfur metabolism, is considered a potential player in the etiology of IBS, inflammatory bowel disease (IBD), and colorectal cancer (Carbonero et al., 2012; Chassard et al., 2012). Taurine, as its important metabolite, can promote the growth of sulfur-producing bacteria and lead to the occurrence of IBD (Walker and Schmitt-Kopplin, 2021). In addition, ABC transporters are key bacterial proteins affecting nutrient absorption and drug resistance (Zhang et al., 2021). Notably, Bile secretion can regulate visceral pain perception and improve visceral hypersensitivity in IBS patients (Ní Dhonnabháín et al., 2021). The increased expression of bile acid in fecal, primary bile acid in liver and bile acid receptor Takeda G protein-coupled receptor 5 (TGR5) in colon in most patients with IBS-D is closely related to the severity of diarrhea symptoms (Walters, 2021; Wei et al., 2021), and the TGR5-ECS-5-HT signaling pathway may play an important role in the pathophysiology of IBS (Tao et al., 2022). Bile acids can control the circadian variation of the metabolite uric acid through the regulation of xanthine oxidase by PPARα (Kanemitsu et al., 2017). In addition, it has been proposed that bile acid malabsorption is caused by changes in intestinal microbiota (Reynaud et al., 2016). Therefore, the interaction between bile acid secretion imbalance and intestinal microbiota can be a potential research point for the pathogenesis of IBS (Fuchs and Trauner, 2022). In conclusion, the discovery of these metabolic pathways is helpful to further clarify the mechanism of SGD in the treatment of IBS, and provide new ideas for the clinical use of SGD in the treatment of IBS patients in the future. In order to better summarize the effect of SGD on intestinal microbiota in IBS-D rats, we drew a graphic summary (Figure 10).

## Data availability statement

The original contributions presented in this study are included in the article/Supplementary material, further inquiries can be directed to the corresponding author.

## Ethics statement

This animal study was reviewed and approved by Animal Ethics Committee of Shanghai University of TCM.

## Author contributions

LH carried out the animal experiments, analyzed the experimental data, and wrote the draft of the manuscript. EW, YF, YZ, YM, and FJ carried out the animal experiments. JY designed the experiments and revised the manuscript. All authors contributed to the article and approved the submitted version.

## Footnotes

1
http://exon.gatech.edu/GeneMark/

2
http://ab.inf.unituebingen.de/software/megan5/

3
https://www.ncbi.nlm.nih.gov/taxonomy
